# EGFR-ERK induced activation of GRHL1 promotes cell cycle progression by up-regulating cell cycle related genes in lung cancer

**DOI:** 10.1038/s41419-021-03721-9

**Published:** 2021-04-30

**Authors:** Yiming He, Mingxi Gan, Yanan Wang, Tong Huang, Jianbin Wang, Tianyu Han, Bentong Yu

**Affiliations:** 1 Department of Thoracic Surgery, The First Affiliated Hospital of Nanchang University, Nanchang, 330006 P. R. China; 2 Jiangxi Institute of Respiratory Disease, The First Affiliated Hospital of Nanchang University, Nanchang, 330006 P. R. China; 3 School of Basic Medical Sciences, Nanchang University, Nanchang, 330031 P. R. China

**Keywords:** Non-small-cell lung cancer, Growth factor signalling

## Abstract

Grainyhead-like 1 (GRHL1) is a transcription factor involved in embryonic development. However, little is known about the biological functions of GRHL1 in cancer. In this study, we found that GRHL1 was upregulated in non-small cell lung cancer (NSCLC) and correlated with poor survival of patients. GRHL1 overexpression promoted the proliferation of NSCLC cells and knocking down GRHL1 inhibited the proliferation. RNA sequencing showed that a series of cell cycle-related genes were altered when knocking down GRHL1. We further demonstrated that GRHL1 could regulate the expression of cell cycle-related genes by binding to the promoter regions and increasing the transcription of the target genes. Besides, we also found that EGF stimulation could activate GRHL1 and promoted its nuclear translocation. We identified the key phosphorylation site at Ser76 on GRHL1 that is regulated by the EGFR-ERK axis. Taken together, these findings elucidate a new function of GRHL1 on regulating the cell cycle progression and point out the potential role of GRHL1 as a drug target in NSCLC.

## Introduction

Grainyhead like transcription factor 1 (GRHL1) is a 74 kDa protein that belongs to the homologous group of GRH families^1^. The Grainyhead genes were originally discovered in a Drosophila mutant^2^, these genes contribute to epidermal barrier formation, trachea elongation, postembryonic neuroblast migration, and the epidermal wound response^3–6^. Recently, many studies have focused on the functions of GRHL in cancer progression. It has been proved that GRHLs played an important role in the occurrence and development of breast cancer, prostate cancer, cervical cancer, and other malignant tumors^1^. However, the studies on GRHL1 mainly focused on embryonic development^7^, little is known about its role in cancer. Research in esophageal squamous cell carcinoma found that GRHL1 was down-regulated in cancer cells and cancer tissues and correlated with a reduced overall survival rate. Overexpressing GRHL1 inhibited the invasion of cancer cells^8^. In colon cancer, GRHL1 expression was higher in tumor tissues than normal colon tissues and the low levels of GRHL1 correlated with better overall survival of cancer patients. In accordance with this, knockdown of GRHL1 inhibited the proliferation of human colon cancer cells^9^. These studies indicated that the functions of GRHL1 were different depending on the types of cancer. In lung cancer, however, the function of GRHL1 has not been studied.

The dysregulation of cell cycle-related genes causes uncontrolled cell cycle progression, leading to unlimited cell proliferation which is one of the hallmarks of cancer. Here, we found a new role of GRHL1 in cell cycle regulation. we showed that NSCLC patient samples and NSCLC cells displayed much higher GRHL1 expression than normal lung tissues or epithelial cells, and the higher expression closely correlated with poor patient survival. We found that GRHL1 promoted the proliferation of NSCLC by regulating the transcription of G2/M phase-related genes: cell division cycle 27 (CDC27), anaphase promoting complex subunit 13 (ANAPC13 or APC13), RAD21 cohesive complex (RAD21) and cell division cycle 7 (CDC7). We also discovered that the EGFR-ERK axis was the upstream signaling pathway for GRHL1 activation and we identified Ser76 as the key phosphorylation site that phosphorylated by ERK. Thus, our findings first demonstrated the function of GRHL1 in NSCLC and elucidated the molecular mechanisms for GRHL1 functioning as an oncogenic gene in NSCLC which provided a new therapeutic strategy for targeting GRHL1 to cure lung cancer.

## Materials and methods

### Reagents

GRHL1 antibody was bought from Sigma-Aldrich (HPA005798). ANAPC13 antibody was bought from Absin (abs132750). CDC7, CDC27, RAD21 antibodies, β-actin antibody were bought from Proteintech (17980-1-AP, 10918-1-AP, 27071-1-AP, 66009-1-lg). Erk1/2 antibody were purchased from CST (4695). Crystal violet and other analytical grade chemicals were purchased from Sigma Aldrich (St. Louis, MO, USA).

### Cell culture

Human bronchial epithelial (HBE) cells were cultured in Airway epithelial cell basal medium using the bronchial/tracheal epithelial cell growth kit (ATCC). Human NSCLC cell lines (H358, H1299, A549, H292, H1975, and SPC-A1) were cultured in RPMI 1640(Gibco) supplemented with 10% FBS (Gibco). Human Renal Epithelial Cells (293 T) were cultured in DMEM (Gibco) supplemented with 10% FBS (Gibco). A549 cells with endogenous GRHL1 knockdown were produced by GRHL1 shRNA Plasmid Kit (ORIGENE: TR304207).

### Luciferase activity assay

Human genomic DNA was extracted from H1299 cells and the RAD21, CDC27, CDC7, ANAPC13 promoter fragments (375 bp, 373 bp, 273 bp, 517 bp) were amplified by PCR and cloned into pGL3-enhancer vector respectively. The pGL3-enhancer vector containing the promoter fragment and Renilla control plasmid were co-transfected into cells using SuperFectin II in vitro DNA transfection reagent (Shanghai Pufei Biotech). Forty-eight hours later, cells were lysed and luciferase activity was detected using the Dual-Luciferase reporter assay kit (Promega). The relative levels of luciferase activity were normalized to the levels of luciferase activity of the Renilla control plasmid.

### Cell cycle analysis

The cells were cultured in 6-well plates with each treatment. Then, the cells were harvested and washed with 1× PBS, followed by adding 70% alcohol to the cells and incubating on ice for 2 h; the cells were washed with 1× PBS and resuspended with 400 µl guava cell cycle reagent (Millipore, 4700–0160) (PI:585/29). After incubation at 37 °C for 15 min, the cells were analyzed using a BD FACS Jazz™ Cell Sorter (Becton Dickinson).

### In vivo xenograft assay

Cell suspensions (1 × 10^7^ cells) in a total volume of 100 μl were injected subcutaneously into the flanks of 3–4-week-old male BALB/C nude mice (SLAC, Shanghai). Four weeks later, the mice were killed. Tumors were dissected out and their weights and volumes were measured. Tumor volume was calculated using the formula: volume (mm^3^) = π/6 × (large diameter) × (smaller diameter)^2^. All mice were housed in the SPF animal facility of the Institute of Translational Medicine at Nanchang University.

### RNA-seq analysis

A549 stable cells with GRHL1 knockdown and wild-type A549 cells were cultured in 15 cm dishes. When the cells were almost spread in culture dishes, digested the cells, centrifuged them, and froze them with liquid nitrogen. The samples were analyzed in the Beijing Genomics institution. Differentially expressed genes were identified through RNA-seq analysis.

### Statistical analysis

All experiments were performed in triplicate. All the data were expressed as the mean ± SD. ANOVA and paired t-tests were used to make statistical comparisons. *P* < 0.05 was considered to be statistically significant.

## Results

### GRHL1 is upregulated in NSCLC tissues and correlates with the poor survival of NSCLC patients

**Fig. 1:**
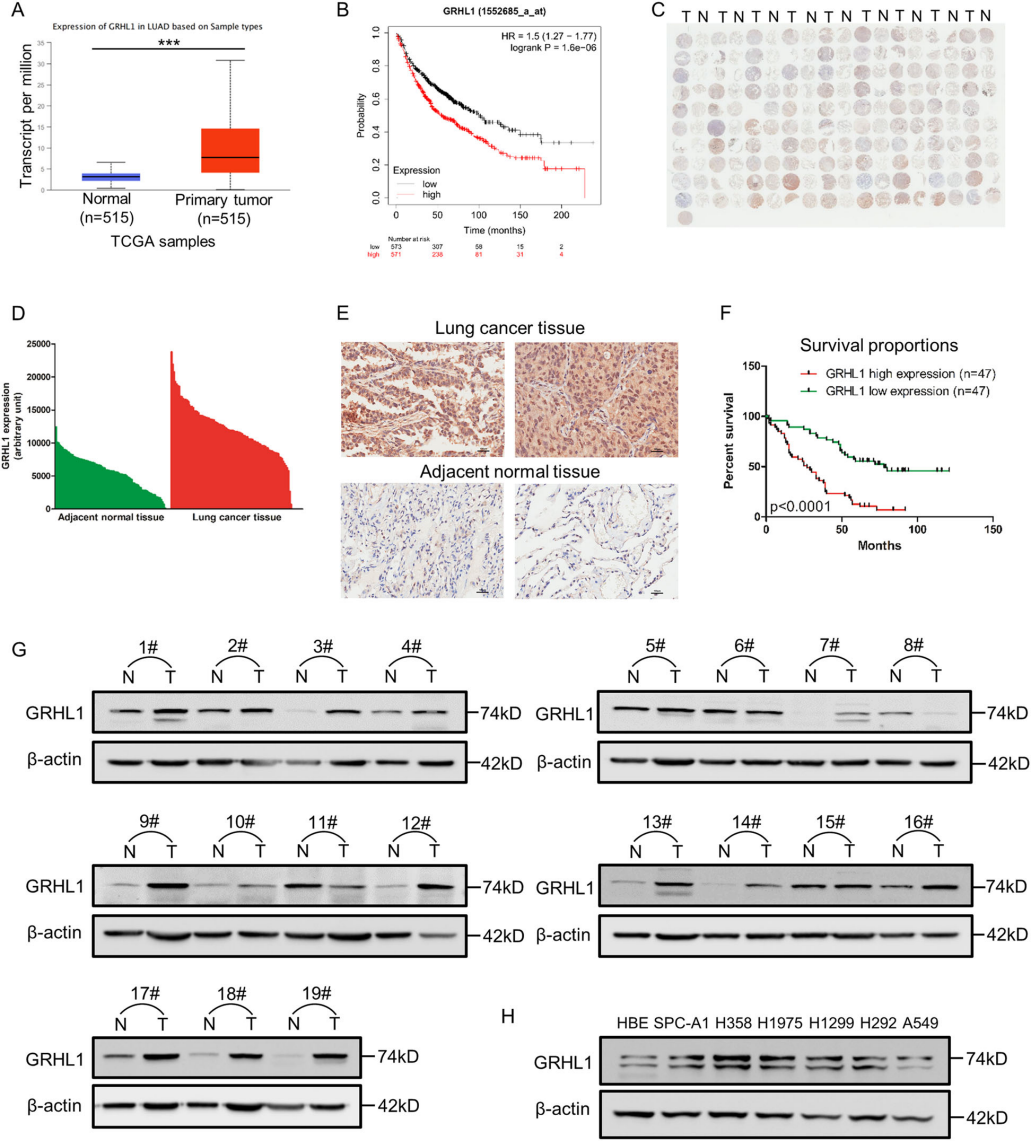
GRHL1 is upregulated in NSCLC and closely related to the poor survival of NSCLC patients. **A** The mRNA levels of NSCLC samples (Normal and Primary tumor) were analyzed from TCGA using the following link: http://ualcan.path.uab.edu/cgi-bin/ualcan-res.pl. ***: *P* < 0.0001. **B** The overall survival curves of NSCLC patients were analyzed using Kaplan–Meier Plotter (https://kmplot.com/analysis/). **C** Immunohistochemical staining of a representative lung adenocarcinoma tissue microarray with an anti-GRHL1 antibody. T, tumor tissue; N, adjacent normal tissue. **D** Quantification of the immunohistochemical (IHC) staining shown in Fig. 1C. **E** Microscopic evaluation of IHC staining of two representative tumor tissues and adjacent normal tissues shown in Fig. 1C with an anti-GRHL1 antibody (brown) and hematoxylin counterstain (blue). Scale bars: 100 μm. **F** Kaplan–Meier survival curve of 94 NSCLC patients. Patients were divided into two groups according to the average staining density of GRHL1 in cancer tissues of the tissue array (high expression: *n* = 47, low expression: *n* = 47, Log-rank (Mantel-Cox) test was used for the statistical analysis). **G** The protein expression levels of GRHL1 were determined by western blot using the paired, tumor-adjacent noncancerous lung tissues (normal, N) and human NSCLC tissues (tumor, T) from 19 NSCLC patients (1#−19#). **H** The expression levels of GRHL1 in NSCLC cell lines (SPC-A1, H358, H1975, H1299, H292, A549) and HBE cells were examined by western blot using an anti-GRHL1 antibody.

To investigate the expression of GRHL1 in NSCLC, we first analyzed the expression of GRHL1 in NSCLC samples in TCGA. The results showed that the mRNA level of GRHL1 is significantly higher in tumor tissues than in normal tissues (Fig. 1A). The relationship between GRHL1 expression and patients’ survival was analyzed using Kaplan–Meier Plotter (https://kmplot.com/analysis/). We discovered that the NSCLC patients with high GRHL1 expression had poorer overall and first progression survival probability than patients with low GRHL1 expression, but there was no significant difference in post progression survival (Fig. 1B and Supplementary Fig. 1A, B). We then purchased an NSCLC tissue microarray and performed immunohistochemistry using an anti-GRHL1 antibody. The tissue microarray contains lung cancer tissue samples and adjacent normal tissue from 94 NSCLC patients. The results showed that 96% of tumor tissues expressed significantly higher levels of GRHL1 than the adjacent normal tissues (Fig. 1C) and this was further validated by quantification of the staining (Fig. 1D). Representative staining of cancer and adjacent normal tissues were presented in Fig. 1E; Expression levels of GRHL1 (brown) were higher in cancer tissues with only weak or no expression observed in adjacent normal tissues. Statistical analysis of the quantified staining results from cancer tissues divided the cancer samples into two groups depending upon the median of GRHL1 expression level (median:11936.5, analyzed by GenePix; high expression: *n* = 47, the low expression: *n* = 47). Survival of the patients was then correlated to the GRHL1 levels. As shown in Fig. 1F, patients with high levels of GRHL1 exhibited poorer overall survival than patients with low levels of GRHL1 (*P* < 0.0001). The multivariate Cox regression analysis model was used to analyze the factors affecting the prognosis and showed that the expression of GRHL1 was an independent predictor of risk in patients with NSCLC (Supplementary Fig. 1C). This indicated that GRHL1 was a poor prognostic factor and an independent prognostic marker. We further analyzed other data of the tissue microarray and found that high expression levels of GRHL1 correlate with the older age and the bigger tumors (Table 1). These results indicated that the GRHL1 was overexpressed in NSCLC tissues and closely related to NSCLC progression and patient survival.

**Table 1:** High expression levels of GRHL1 correlate with the older patients and the bigger tumors.

Factor	number	Mean rank	*p* vaule
AJCC stage			
I	30	11987.3	–
II	20	11703.95	–
II-III	13	14088.31	ns
III	28	11890.93	–
IV	1	11151	–
Tumor volume			
<36 cm³	47	11494.02	–
≥36 cm³	47	12887.45	<0.05
Age at diagnosis			
20–57	34	11147.29	–
58–84	60	12782.02	<0.05

GRHL1 expression values were acquired from a representative lung adenocarcinoma tissue microarray of 94 adenocarcinoma samples. One-tailed *t* test was used for the comparison of GRHL1 expression between different subgroups.

To further investigate the expression of GRHL1 in NSCLC, we examined GRHL1 protein expression in tumor and adjacent normal tissues from 19 NSCLC patients. The results demonstrated that the expression of GRHL1 was higher in most tumor tissues than that in normal lung tissues (Fig. 1G). Then we checked GRHL1 protein expression in human bronchial epithelial cells (HBE) and NSCLC cell lines. The NSCLC cell lines exhibited higher GRHL1 expression than HBE cells (Fig. 1H). These results further suggested that GRHL1 was overexpressed in both NSCLC tissue samples and NSCLC cell lines.

### GRHL1 promotes the proliferation of NSCLC cells

**Fig. 2:**
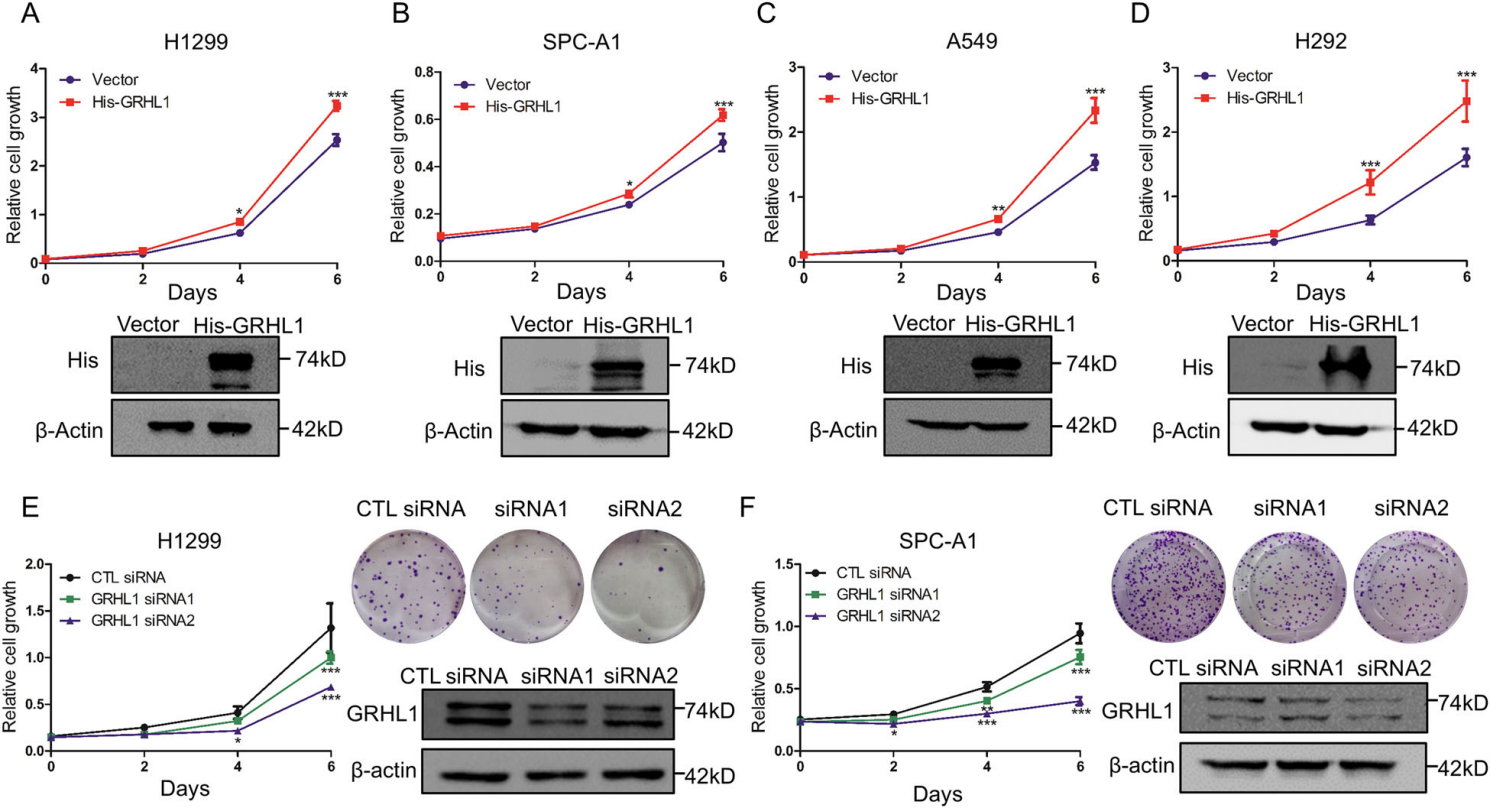
GRHL1 promotes the proliferation of NSCLC cells. **A–D** H1299 (**A**) cells, SPC-A1 (**B**) cells, A549 (**C**) cells, and H292 (**D**) cells were transfected with pcDNA3.1 plasmid or pcDNA3.1-His-GRHL1 (His-GRHL1) plasmid. At the indicated times, cells were fixed in 3.7% formaldehyde and stained with 0.1% crystal violet. Data represent the average of three independent experiments (mean ± SD). *P < 0.05, ***P < 0.001. Western blot was used to examine the expression of the indicated proteins. **E, F** H1299 (**E**) and SPC-A1 (**F**) cells were transfected with control (CTL) siRNAs or GRHL1 siRNAs. At the indicated times, cells were fixed in 3.7% formaldehyde and stained with 0.1% crystal violet. Data represent the average of three independent experiments (mean ± SD). *P < 0.05, **P < 0.01, ***P < 0.001. For colony formation assay, H1299 and SPC-A1 cells were transfected with control siRNA or siRNAs targeting GRHL1. Eight days later, colony formation was detected by crystal violet staining and photographed. Protein expression was assessed by western blot using indicated antibodies.

To determine the biological function of GRHL1 in NSCLC cells, we performed cell proliferation assay. As shown in Fig. 2A–D, overexpressing GRHL1 significantly promoted the proliferation of NSCLC cells, indicating the oncogenic function of GRHL1. To further confirm the results, GRHL1 was knocked down using GRHL1 specific siRNAs, followed by proliferation assays. As shown in Fig. 2E, F, GRHL1 knockdown dramatically inhibited the proliferation and colony formation of NSCLC cells. These results suggested that GRHL1 promoted the proliferation of NSCLC cells.

### GRHL1 knockdown influences the cell cycle pathway and arrests the cell cycle in the G2/M phase

**Fig. 3:**
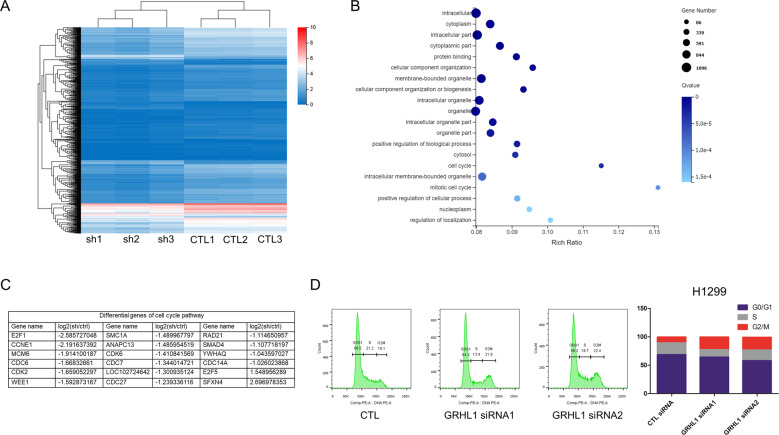
GRHL1 knockdown influences cell cycle progression and arrests the cell cycle in the G2/M phase. **A**–**C** RNA-sequencing. The samples using wild-type A549 (CTL) and A549-shGRHL1 (sh) cell lines, CTL and shGRHL1 groups contain 3 repeat groups respectively. The samples were sequenced in BGI (Beijing Genomics institution). **A** Cluster heat map of 1379 differentially expressed genes regulated by GRHL1. The horizontal axis represents log2(FPKM + 1) of samples, and the vertical axis represents genes. The redder color indicated the higher expression level and the bluer color indicated the lower expression level. **B** GO enrichment bubble diagram: according to the results of GO annotation and official classification, classified the differential genes functionally and conducted enrichment analysis. **C** The differential genes of cell cycle pathway. **D** H1299 cells were transfected with either control (CTL) siRNA or GRHL1 siRNAs. After 24 h, cell cycle analysis was done by flow cytometry. The inserts show the quantification of the cell cycle analysis.

To explore the molecular mechanism of GRHL1 in regulating cell proliferation, we performed RNA sequencing. The results showed that GRHL1 knockdown generated 1379 differentially expressed genes, as shown in the Cluster heat map of differential gene expression (Fig. 3A). We further conducted a Gene Ontology analysis, the result indicated that GRHL1 knockdown significantly influenced several pathways, especially cell cycle and mitotic cell cycle pathways, which have higher rich ratios (Fig. 3B). The differential genes related to the cell cycle are shown in Fig. 3C. To further verify the effect of GRHL1 on cell cycle progression in NSCLC cells, we conducted flow cytometry. As can be seen in Fig. 3D and Supplementary Fig. 2A, knockdown of GRHL1 obviously influenced cell cycle progression and arrested cell cycle at the G2/M phase. These results demonstrated that GRHL1 was indispensable for cell cycle progression. Thus, we speculated that GRHL1 might promote the proliferation of NSCLC cells by regulating the transcription of cell cycle-related genes.

### GRHL1 regulates the transcription of genes in the G2/M phase

**Fig. 4:**
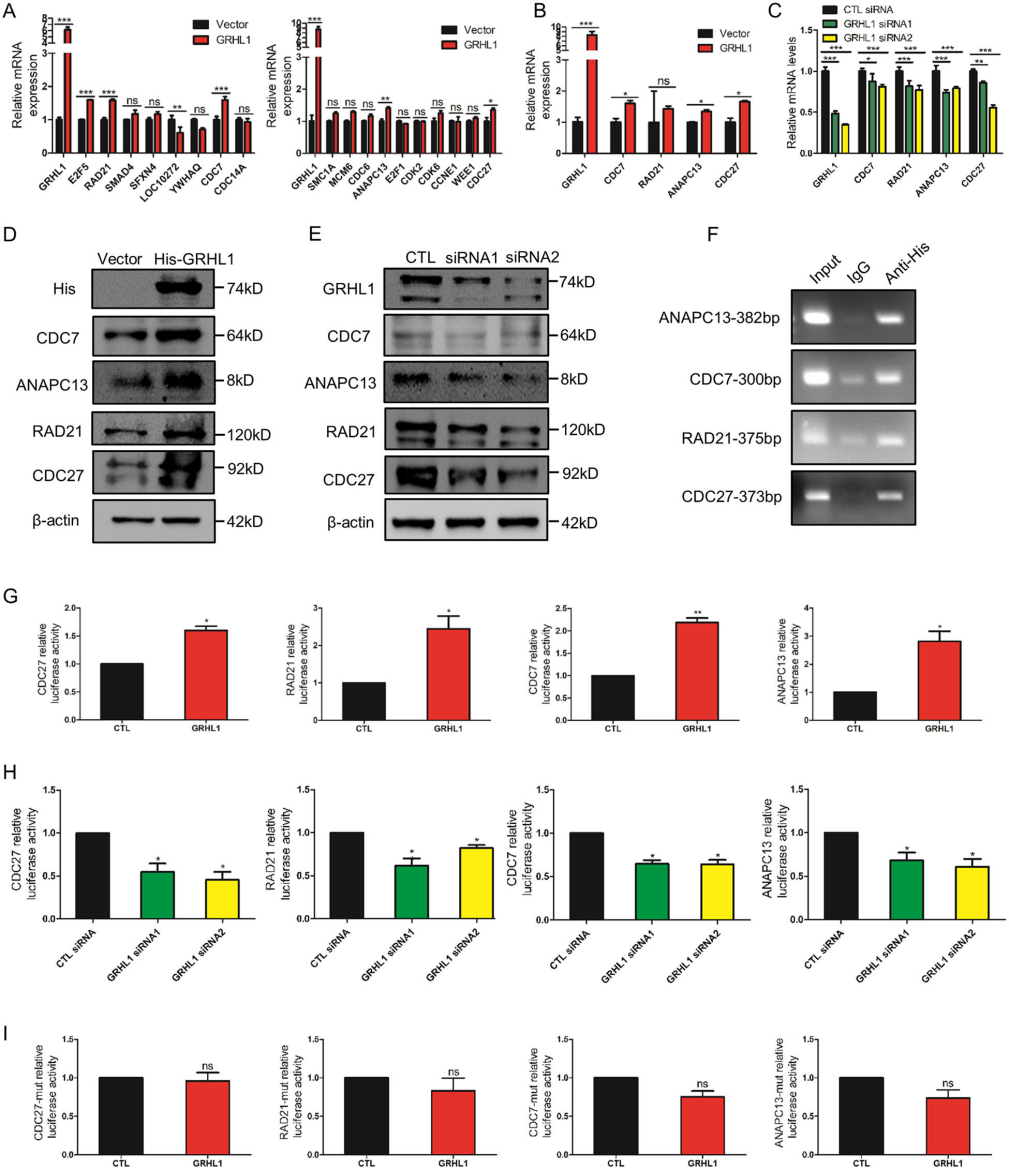
GRHL1 regulates the transcription of genes in the G2/M phase. **A**, **B** H1299 cells were transiently transfected with control or GRHL1 plasmids. Forty-eight hours later, total RNAs were extracted. The mRNA levels were determined by q-PCR. Data represent the average of three independent experiments. *P < 0.05, **P < 0.01, ***P < 0.001, ns: *P* > 0.05. **C** H1299 cells were transiently transfected with control or GRHL1 siRNAs. Forty-eight hours later, total RNAs were extracted. The mRNA levels were determined by q-PCR. Data represent the average of three independent experiments. *P < 0.05, **P < 0.01, ***P < 0.001. **D** H292 cells were transiently transfected with control or His-GRHL1 plasmids. Forty-eight hours later, the cells were lysed. Protein expression was assessed by western blotting using the indicated antibodies. **E** H1299 cells were transiently transfected with control or GRHL1 siRNAs. Forty-eight hours later, the cells were lysed. Protein expression was assessed by western blotting using the indicated antibodies. **F** In 293 T cells, ChIP assay was conducted with anti-His and control rabbit IgG for immunoprecipitation, followed by PCR with CDC27, RAD21, CDC7, and ANAPC13 promoter-specific primers. **G** pGL3-enhancer vector containing CDC27, RAD21, CDC7, and ANAPC13 promoter fragment was transfected into H1299 respectively, co-transfected with Renilla control plasmid and pcDNA3.1 vector or pcDNA3.1-GRHL1 plasmid. The relative levels of luciferase activity were normalized to the levels of vector control and to the levels of luciferase activity of the Renilla control plasmid. Data represent the average of three independent experiments (mean ± SD). *P < 0.05, **P < 0.01. **H** pGL3-enhancer vector containing CDC27, RAD21, CDC7, and ANAPC13 promoter fragment was transfected into H1299 respectively, co-transfected with Renilla control plasmid and control siRNA or GRHL1 siRNAs. The relative levels of luciferase activity were normalized to the levels of vector control and the levels of luciferase activity of the Renilla control plasmid. Data represent the average of three independent experiments (mean ± SD). *P < 0.05. **I** pGL3-enhancer vector containing CDC27-mutation, RAD21-mutation, CDC7-mutation, and ANAPC13-mutation promoter fragment was transfected into H1299 respectively, co-transfected with Renilla control plasmid and pcDNA3.1 vector or pcDNA3.1-GRHL1 plasmid. The relative levels of luciferase activity were normalized to the levels of vector control and the levels of luciferase activity of the Renilla control plasmid. Data represent the average of three independent experiments (mean ± SD). ns *P* > 0.05.

As GRHL1 is a transcription factor, we next examined if GRHL1 regulated the transcription of cell cycle-related genes. First, we overexpressed GRHL1 in H1299 and A549 cells followed by examining the mRNA expression of cell cycle-related genes in Fig. 3C. Overexpression of GRHL1 increased the mRNA levels of CDC27, RAD21, CDC7, and ANAPC13 which played important roles in G2/M phase (Fig. 4A and Supplementary Fig. 3A). This result was consistent with our RNA-seq results. To further verify this, GRHL1 was overexpressed or knocked down and the mRNA expressions of CDC27, RAD21, CDC7, and ANAPC13 were examined. Overexpressing GRHL1 increased the mRNA levels of these genes, while knockdown of GRHL1 decreased their mRNA expression (Fig. 4B-C and Supplementary Fig. 3B). Then we examined the protein expression of these genes. As shown in Fig. 4D-E and Supplementary Fig. 3C-D, overexpressing GRHL1 increased the protein expression of CDC27, RAD21, CDC7, and ANAPC13, while knockdown of GRHL1 significantly decreased the protein expression. These results suggested that CDC27, RAD21, CDC7, and ANAPC13 might be the potential target genes of GRHL1. Analysis of the promoter region of these genes identified putative GRHL1 binding sequences: AACTAGTT of CDC27, TACACGTT of RAD21, AACATGTT of CDC7, and GACACGTC of ANAPC13. Chromatin immunoprecipitation showed that GRHL1 could bind to the promoter regions of CDC27, RAD21, CDC7, and ANAPC13 (Fig. 4F). We then cloned the promoter regions of these genes into the pGL3-enhancer vector respectively and performed luciferase assays. We found that the promoter activity of these genes increased dramatically when GRHL1 was overexpressed (Fig. 4G and Supplementary Fig. 3E) but decreased when GRHL1 was knocked down (Fig. 4H and Supplementary Fig. 3F). As the first three bases of the binding sequences are relatively conservative, so we mutated AACTAGTT, TACACGTT, AACATGTT, GACACGTC into CTGTAGTT, CTGACGTT, CTGATGTT, CTGACGTC respectively, and conducted luciferase assays. The promoter activity of CDC27-mutation, RAD21-mutation, CDC7-mutation, and ANAPC13-mutation did not increase when GRHL1 was overexpressed (Fig. 4I). These data indicated that GRHL1 could bind to the promoter regions of CDC27, RAD21, CDC7, and ANAPC13 and activate their transcription.

### EGFR-ERK pathway activates GRHL1 and promotes its nuclear translocation

**Fig. 5:**
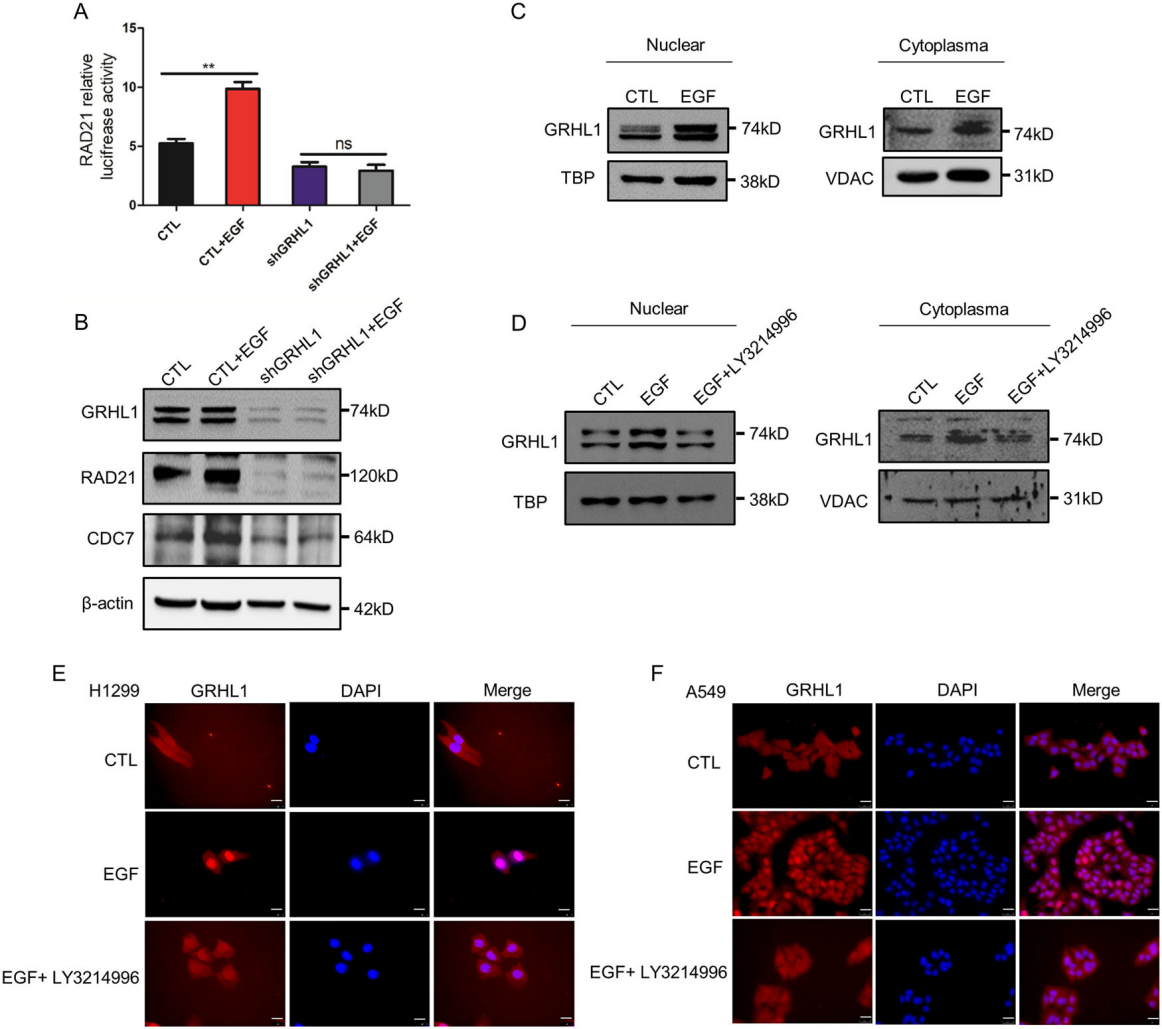
EGFR-ERK pathway activates GRHL1 and promotes its nuclear translocation. **A** pGL3-enhancer vector containing RAD21 promoter fragment was transfected into A549-control cells and A549-shGRHL1 cells, co-transfected with Renilla control plasmid. After serum-free treatment for 12 h, EGF (100 ng/ml) was added to two groups to stimulate for 24 h. The relative levels of luciferase activity were normalized to the levels of luciferase activity of the Renilla control plasmid. Data represent the average of three independent experiments (mean ± SD). **P < 0.01. **B** After serum-free treatment for 12 h of A549-control cells and A549-shGRHL1 cells, EGF (100 ng/ml) was added to two experimental groups. Twenty-four hours later, the cells were lysed. Protein expression was assessed by western blotting using the indicated antibodies. **C** After 24 h of stimulation with EGF (100 ng/ml), the nuclear and cytoplasmic fractions were separated and the location of GRHL1 was detected by western blot in A549 cells. TBP: TATA binding protein, VDAC: mitochondrial outer membrane protein porin. **D** After serum-free treatment for 12 h of A549 cells, LY3214996 (ERK inhibitor, 300 nM) was added. 12 h later, EGF (100 ng/ml) was added to both two experimental groups. After 24 h, the nuclear and cytoplasmic fractions were separated and the location of GRHL1 was detected by western blot. **E, F** After serum-free treatment for 12 h of A549 and H1299 cells, LY3214996 (ERK inhibitor, 300 nM) was added. 12 h later, EGF (100 ng/ml) was added to both two experimental groups. After 24 h. The location of GRHL1 was detected by immunofluorescence staining. Cells were stained with anti-GRHL1 (left panel, red) and DAPI (middle panel, blue). The merged images are shown in the right panel. Scale bar = 25 µm, magnification: ×400.

The EGFR pathway plays a vital role in tumor proliferation. The dimerization of EGFR can activate important pathways such as the ERK pathway, thereby activating a variety of transcription factors and inducing the proliferation and differentiation of tumor cells. Thus, we wanted to explore if GRHL1 could be regulated by the EGFR pathway. Using luciferase assay, we found that EGF stimulation dramatically increased the activity of the RAD21 promoter, but this effect disappeared when GRHL1 was knocked down (Fig. 5A). We also found that EGF stimulation did not change the expression of GRHL1 but increased the protein levels of its target genes RAD21 and CDC7, and knocking down GRHL1 diminished this effect (Fig. 5B). Besides, EGF stimulation significantly promoted the nuclear translocation of GRHL1 (Fig. 5C and Supplementary Fig. 4A). However, when the cells were pretreated with ERK inhibitor LY3214996, EGF stimulation did not increase the nuclear translocation of GRHL1 (Fig. 5D-F and Supplementary Fig. 4B). These results demonstrated that the EGFR-ERK pathway was the upstream regulator of GRHL1.

### ERK is responsible for the phosphorylation of GRHL1 at Ser76

**Fig. 6:**
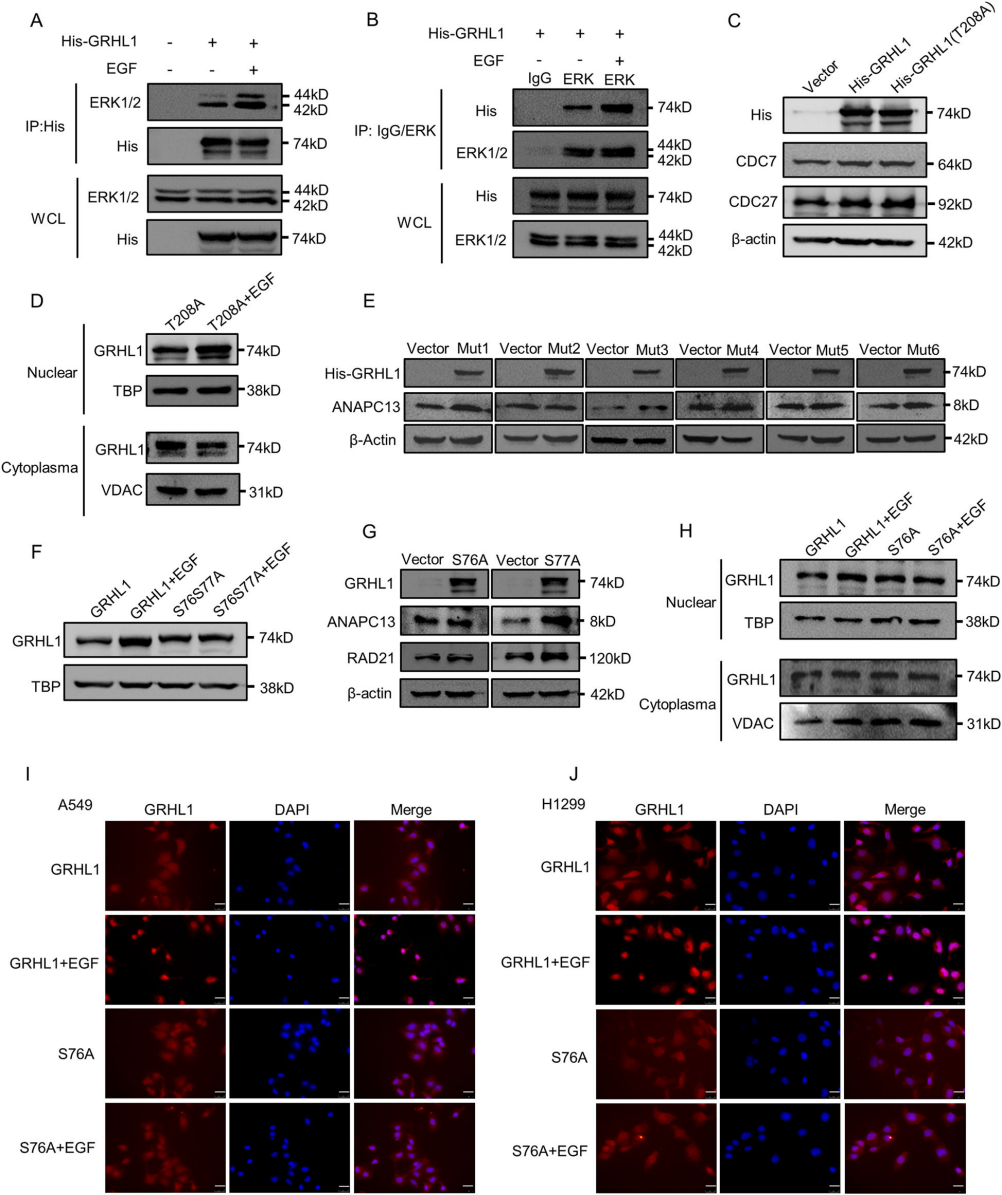
ERK is responsible for the phosphorylation of GRHL1 at Ser76. **A** 293 T cells were transfected with control plasmid and His-GRHL1 plasmid. After serum-free treatment for 12 h and 12 h of stimulation with EGF (100 ng/ml), cells were lysed for immunoprecipitation using an anti-His antibody and blotted with indicated antibodies. **B** 293 T cells were transfected with His-GRHL1. After serum-free treatment for 12 h and 12 h of stimulation with EGF (100 ng/ml), cells were lysed for immunoprecipitation using anti-ERK1/2 antibody and control rabbit IgG and blotted with indicated antibodies. **C** A549 cells were transiently transfected with control plasmid, His-GRHL1 plasmid and His-GRHL1(T208A) plasmid respectively. Forty-eight hours later, the cells were lysed. Protein expression was assessed by western blotting using the indicated antibodies. **D** A549 cells were transfected with His-GRHL1(T208A) plasmid, after serum-free treatment for 12 h and 24 h of stimulation with EGF (100 ng/ml), the nuclear and cytoplasmic fractions were separated and the location of GRHL1 was detected by western blot. **E** A549 cells were transiently transfected with control plasmid and six mutants (mut1: S25S28S35A, mut2: S76S77A, mut3: S95A, mut4: S442A, mut5: S493S501A, mut6: S539A) plasmids respectively. Forty-eight hours later, the cells were lysed. Protein expression was assessed by western blotting using the indicated antibodies. **F** A549 cells were transfected with His-GRHL1 plasmid and His-GRHL1(S76S77A) plasmid, after serum-free treatment for 12 h and 24 h of stimulation with EGF (100 ng/ml), the nuclear and cytoplasmic fractions were separated and the GRHL1 in nuclear was detected by western blot. **G** A549 cells were transiently transfected with control plasmid, His-GRHL1(S76A), and His-GRHL1(S77A) plasmids respectively. Forty-eight hours later, the cells were lysed. Protein expression was assessed by western blotting using the indicated antibodies. **H** A549 cells were transfected with His-GRHL1 plasmid and His-GRHL1(S76A) plasmid, after serum-free treatment for 12 h and 24 h of stimulation with EGF (100 ng/ml), the nuclear and cytoplasmic fractions were separated and the location of GRHL1 was detected by western blot. **I**–**J** A549 and H1299 cells were transfected with His-GRHL1 plasmid and His-GRHL1(S76A) plasmid. After serum-free treatment for 12 h, EGF (100 ng/ml) was added. After 12 h. The location of GRHL1 was detected by immunofluorescence staining. Cells were stained with anti-GRHL1 (left panel, red) and DAPI (middle panel, blue). The merged images are shown in the right panel. Scale bar = 25 µm, magnification: ×400.

To determine whether ERK could directly phosphorylate GRHL1, we performed co-immunoprecipitation experiments. We found ERK could interact with GRHL1 and when stimulated with EGF, the interaction was obviously enhanced (Fig. 6A, B). To find out the phosphorylation site, mass spectrometric analyses were conducted and a phosphorylation site at Thr208 of GRHL1 was found (Supplementary Table 2). We mutated Thr208 into alanine and transfected the mutant or wild-type His-GRHL1 into A549 and H1299 cells, followed by assessing the expression of target genes. The results demonstrated that mutation of Thr208 to Ala (T208A) had a similar effect as GRHL1 wild-type on the expression of target genes (Fig. 6C and Supplementary Fig. 5A). Besides, the nuclear translocation of T208A could still be promoted by EGF stimulation (Fig. 6D and Supplementary Fig. 5B). This indicated that Thr208 might not be the key phosphorylation site regulated by ERK. Then we used the PhosphoSitePlus program to get the potential phosphorylation information of GRHL1. The program showed 10 phosphorylation sites of GRHL1 (T208, Ser25, Ser28, Ser35, Ser76, Ser77, Ser95, Ser442, Ser493, Ser501, and Ser539). We then mutated these residues into alanines, transfected the mutants into A549 or H1299 cells, and assessed the expression of ANAPC13. Mutation of serine 76 and 77 to alanine (mut2: S76S77A) did not enhance the expression of ANAPC13 while the other five mutants increased the ANAPC13 expression (Fig. 6E and Supplementary Fig. 5C). Moreover, the nuclear translocation of the S76S77A mutant could not be promoted by EGF stimulation (Fig. 6F and Supplementary Fig. 5D). These results indicated that one or two of these two sites might be the key phosphorylation site regulated by ERK. We further mutated Ser76 and Ser77 respectively into alanine. As shown in Fig. 6G and Supplementary Fig. 5E, mutant S77A could still increase the expression of RAD21 and CDC7, while S76A lost this function. And the nuclear translocation of the S76A mutant could not be promoted by EGF stimulation (Fig. 6H-J and Supplementary Fig. 5F). Moreover, the S76A mutant did not affect the interaction between ERK1/2 and GRHL1 (Supplementary Fig. 5G). All these results demonstrated that S76A was the key phosphorylation site that mediated the translocation of GRHL1 into nuclear and promoting the transcription of target genes.

### GRHL1 knockdown inhibits the tumor growth in the xenograft model

**Fig. 7:**
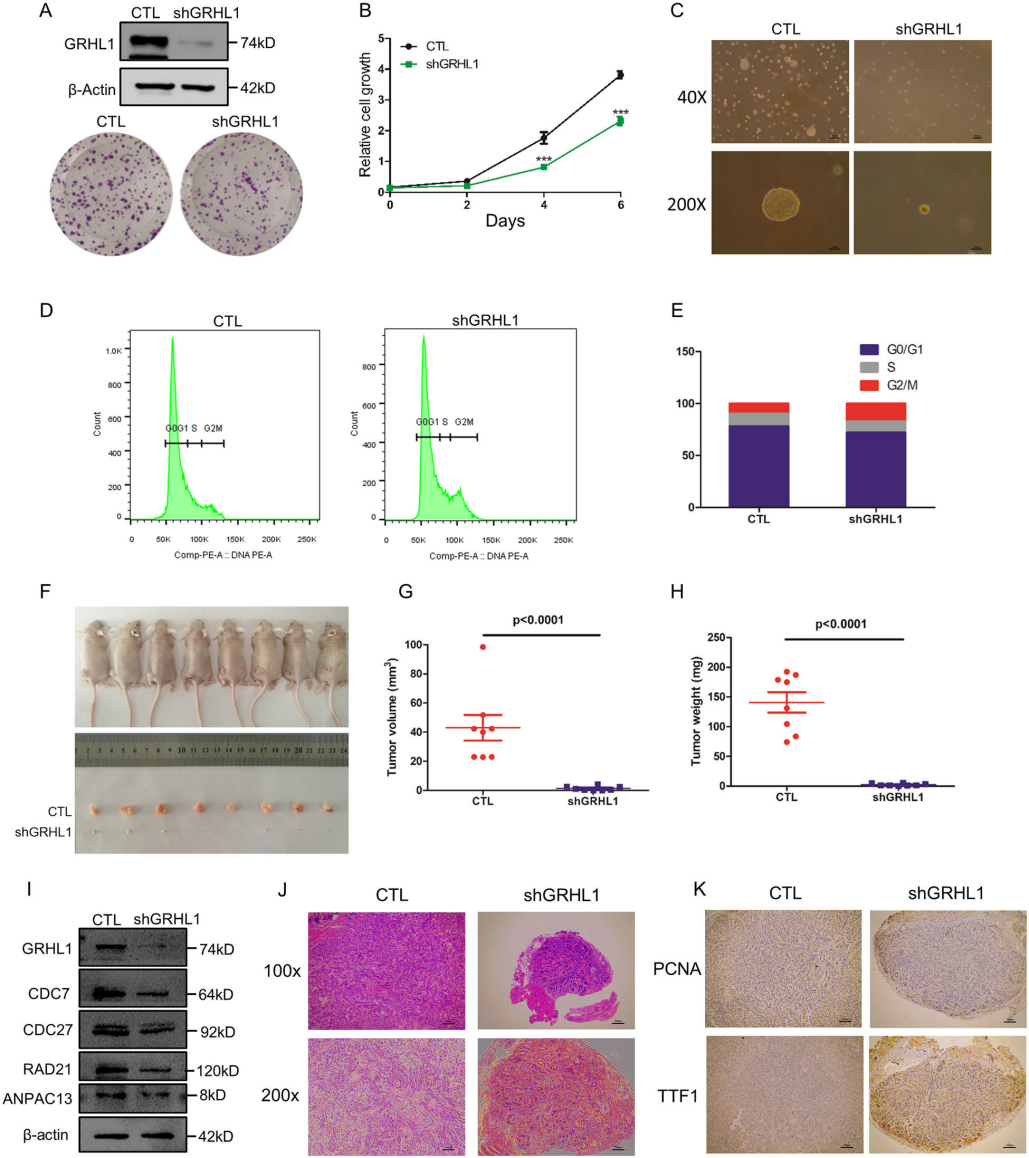
GRHL1 knockdown inhibits the tumor growth in the xenograft model. **A** Upper panels: The knockdown efficiency of A549 stable cell lines detected by western blots. Bottom panels: A549-WT and A549-shGRHL1 cells were trypsinized, counted, and seeded in 6-well plates with 500 cells per well. After 10 days, cells were fixed and stained with crystal violet. Representative wells were photographed and shown. **B** A549-WT and A549-shGRHL1 cells were cultured in RPMI 1640 with 10% FBS for indicated times, and then cells were fixed in 3.7% formaldehyde and stained with 0.1% crystal violet. Dye was extracted with 10% acetic acid and the relative proliferation was determined by the absorbance at 595 nm. Data represent the average of three independent experiments (mean ± SD). ***P < 0.001. **C** Soft agar assay. A549-WT cells or A549-shGRHL1 stable cells were suspended with RPMI 1640 supplemented with 10% FBS and 0.3% agarose followed by plating on top of a solidified layer of RPMI 1640 supplemented with 10% FBS and 0.5% agarose. Cultivated for 2 weeks. The photographs of colonies in soft agar assay. Magnification of upper figures is ×40, scale bars: 400 μm. The magnification of lower figures is ×200, scale bars: 100 μm. **D**, **E** A549-WT and A549-shGRHL1 cells were cultured in RPMI 1640 with 10% FBS for the indicated time, cell cycle analysis was done by flow cytometry. The inserts show the quantification of the cell cycle analysis. **F**–**J** Wide-type A549 cells and A549-shGRHL1 mutant cells (1 × 10^7^) were subcutaneously injected into the flanks of nude mice. Four weeks later, tumors were dissected out, photographed (**F**), and their weights and volumes measured. The *p* value was calculated by paired *t* test (**G**, **H**). The xenograft tumors was lysed. Protein expression was assessed by western blotting using the indicated antibodies(**I**). Photomicrographs of hematoxylin-eosin (HE) staining for tumors induced by wide-type A549 cells and A549-shGRHL1 cells. Magnification of upper figures is ×100, scale bars: 200 μm. The magnification of lower figures is ×200, scale bars: 100 μm(**J**). **K** Immunohistochemical staining in tumors induced by wide-type A549 cells and A549-shGRHL1 cells for PCNA and TTF1. Magnification is ×200, scale bars: 100 μm.

To assess the effect of GRHL1 on tumor growth in vivo, we generated an A549 stable cell line with GRHL1 knockdown. The knockdown efficiency was detected by Western blot (Fig. 7A, upper panels). The proliferation, colony formation, and anchorage-independent growth ability of the A549-shGRHL1 cell line were dramatically inhibited (Fig. 7A–C). And the cell cycle of the A549-shGRHL1 cell line was arrested at the G2/M phase (Fig. 7D, E). We then assessed the tumorigenic ability of A549-shGRHL1 cells in a xenograft model. We observed that xenografts of A549-shGRHL1 cells displayed a dramatically reduced tumor size and weight compared with wide-type A549 cells (Fig. 7F–H). In the xenograft tumors, the expression of GRHL1, CDC27, RAD21, CDC7, and ANAPC13 was significantly decreased (Fig. 7I). HE staining showed that the tumors arising from the wide-type A549 cells were heterogeneous in cell size and had many giant cells, accompanied by disordered nucleo-cytoplasmic ratios and pathological fission, indicative of very poor differentiation (Fig. 7J). However, the structure of the adenoid gland could still be easily observed in tumors formed by A549-shGRHL1 cells and they were much more uniform in size with morphologies suggestive of a high degree of differentiation (Fig. 7J). To confirm these results, we detected the expression of genes indicative of the differentiation states of lung adenocarcinoma by immunohistochemistry. PCNA is widely used as a proliferation marker in pathological assessments. We found that the tumors formed by wide-type A549 cells were PCNA-positive, whereas tumors derived from A549-shGRHL1 cells were PCNA-negative (Fig. 7K, upper panels). NK2 homeobox 1, also known as thyroid TF-1 (TTF-1), was demonstrated to be frequently suppressed in high-grade lung adenocarcinoma. The tumors formed by wide-type A549 cells were TTF1-negative, whereas TTF1-positive cells were observed in tumors formed by A549-shGRHL1 cells (Fig. 7K, bottom panels). These results indicated that the inhibition of GRHL1 promoted lung cancer differentiation.

## Discussion

Previous studies showed that the GRHL family could function as both transcriptional activator and transcriptional inhibitor. Through whole-genome ChIP-seq analysis, it was found that GRHLs mainly functioned as a transcriptional activator in proliferating cells, indicating its potential role in cancer cell proliferation^10^. Now it has been proved that GRHLs played an important role in the occurrence and development of breast cancer, prostate cancer, cervical cancer and other malignant tumors^1^. In different types of tumors, the role of GRHL protein was also complex. In breast cancer, GRHL2 down-regulated Smad and ZEB1 expression thus inhibited TGF-β-induced tumor metastasis and ultimately limited the cancer progression^11^. On the contrary, GRHL2 was highly expressed in liver cancer tissues, and its expression level was closely related to the tumor size, differentiation degree, TNM stage, and positively correlated with the tumor deterioration degree^12^. In skin squamous cell carcinoma, GRHL3 functioned as a tumor suppressor gene by activating PTEN expression^13^. However, in breast cancer, the expression level of GRHL3 was significantly increased and positively correlated with the degree of tumor deterioration^14^. Therefore, exploring the function of GRHL genes in tumors requires specific analysis of tissue types. However, the mechanisms of GRHL in controlling gene expression and its participation in cancer cell proliferation is undefined.

The study of GRHL1 has focused on embryonic development and there are few studies of GRHL1 in cancer research. GRHL1 was reported to involve in the occurrence and development of skin squamous cell carcinoma and neuroblastoma. GRHL1 knockout in mice could damage the skin barrier, which is related to the occurrence of skin squamous cell carcinoma^15^. But in colon cancer, GRHL1 promoted the proliferation of colon cancer^9^. This indicated that the functions of GRHL1 were different depending on the types of cancer. However, the role of GRHL1 in other tumors and its functional mechanism are still unknown.

In our study, we demonstrated that GRHL1 had higher expression in NSCLC samples and correlated with poor survival of NSCLC patients. GRHL1 promoted cell growth and cell cycle progression. Through RNA-sequencing, we discovered that GRHL1 could regulate the transcription of the genes in the G2/M phase. This is the first study demonstrating that GRHL1 could regulate cell cycle-related genes. As we know, transcription factors are the effectors of signaling pathways, but the upstream regulator of GRHL1 is still unclear. Another important finding of our study was that we elucidated the upstream regulators of GRHL1. We demonstrated that EGF stimulation activated and promoted the nuclear translocation of GRHL1. We also identified phosphorylation at Ser76 was responsible for the activation of GRHL1 which was mediated by ERK. Taken together, our study unveiled the molecular mechanism of GRHL1 in regulating cell cycle progression and clarified the upstream signaling pathway that activated GRHL1, which provided a theoretical basis for GRHL1 as a potential drug target for NSCLC treatment.

## Supplementary information

Supplementary information

Supplementary Table 1

Supplementary Table 2
