# Author Correction: EGFR-ERK induced activation of GRHL1 promotes cell cycle progression by up-regulating cell cycle related genes in lung cancer

**DOI:** 10.1038/s41419-024-07055-0

**Published:** 2024-11-04

**Authors:** Yiming He, Mingxi Gan, Yanan Wang, Tong Huang, Jianbin Wang, Tianyu Han, Bentong Yu

**Affiliations:** 1 Department of Thoracic Surgery, The First Affiliated Hospital of Nanchang University, Nanchang, 330006 P. R. China; 2 Jiangxi Institute of Respiratory Disease, The First Affiliated Hospital of Nanchang University, Nanchang, 330006 P. R. China; 3 School of Basic Medical Sciences, Nanchang University, Nanchang, 330031 P. R. China

Correction to: *Cell Death and Disease* 10.1038/s41419-021-03721-9, published online 30 April 2021

In this article the bands for GRHL1 and β-actin in the western blot of Fig. 2F contained errors and have been replaced with the correct bands.

Amended Figure 2
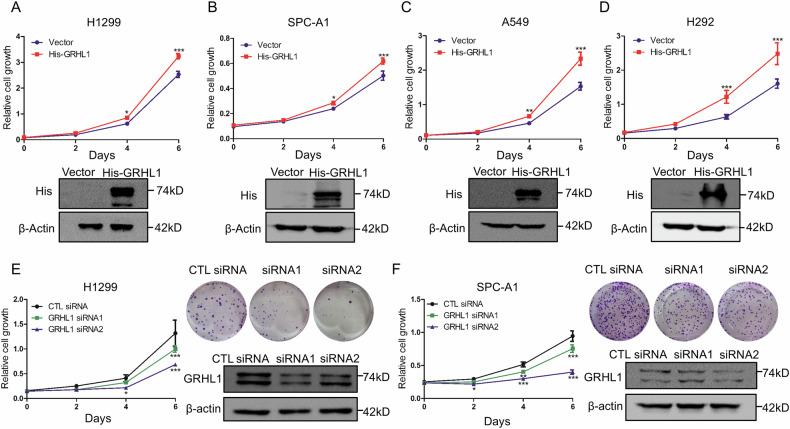


Original Figure 2
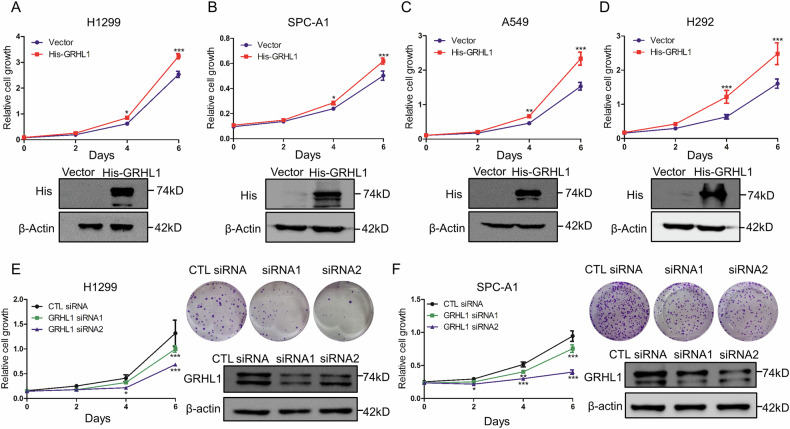


The original article has been corrected.

