# Supplementary material for: EGFR-ERK induced activation of GRHL1 promotes cell cycle progression by up-regulating cell cycle related genes in lung cancer
Source: Cell Death Dis. 2021 Apr 30;12(5):430. doi: 10.1038/s41419-021-03721-9 (PMC8087693; doi:10.1038/s41419-021-03721-9)
Supplement: Supplementary file 1 — Supplementary information [file 41419_2021_3721_MOESM1_ESM.docx]

**Supplementary information**

**Supplementary Materials and Methods**

**Cell growth assay**

For cell proliferation assay with forced or knocked down GRHL1 expression, cells were seeded in 24-well plates at 3000 cells per well in 0.5 ml medium with 10% FBS. On the following day, the medium was changed to RPMI 1640 with different treatments. The medium was changed every 2 days. At the indicated time points, cells were fixed in 4% formaldehyde and stained with 0.1% crystal violet. Dye was extracted with 10% acetic acid and the relative proliferation was determined by the absorbance at 595 nm.

For soft agar assays, 10^4^ cells were suspended in RPMI 1640 supplemented with 10% FBS and 0.3% agarose followed by plating on a solidified layer of RPMI 1640 supplemented with 0.5% agarose and 10% FBS. Fresh medium with 10% FBS and 0.5% agarose were added to the cells every week. Two weeks later, colonies larger than 50 μM were scored.

**Colony formation**

Five hundred cells were seeded in 6-well dishes in RPMI-1640 with 10% FBS. After 10 days of culture, cells were fixed with 4% formaldehyde and stained with 2% crystal violet. The images were obtained using a digital camera (Canon, EOS70D).

**DNA constructs**

The promoter region of CDC27, RAD21, ANAPC13, and CDC7 were obtained by PCR using the gDNA library from NCI-H1299 cells. The construct was cloned into pGL3-Enhancer vector in Xho I and Kpn I restriction sites and examined by sequencing.

**Plasmids and siRNA**

The pCDNA3.1-His-GRHL1 plasmid and mutants of GRHL1 (T208A, S25S28S35A, S76S77A, S95A, S442A, S493S501A, S539A) plasmid were ordered from TSINGKE. The PGL3-enhancer plasmid of ANAPC13-mutation, CDC7-mutation, RAD21-mutation, CDC27-mutation were ordered from TSINGKE. GRHL1 shRNA Plasmid (Cat.TR304207) was purchased from ORIGENE. The siRNAs targeting GRHL1(HSS120977, HSS120978, HSS179071) were purchased from Thermo Fisher (Cat.#1299001). HSS120977 and HSS120978 were used for knocking down GRHL1. NSCLC cell lines were transiently transfected with the indicated siRNAs or Stealth RNAi^TM^ siRNA Negative Control using Superfectin II In Vitro siRNA Transfection Reagent (Shanghai Pufei Biotech). The knockdown efficiency was determined by western blot using indicated antibodies.

**Patient samples**

Fresh specimens of human non-small cell lung cancer and paired normal tissues were supplied by the Department of Thoracic Surgery from the First Affiliated Hospital of Nanchang University. All specimens were collected with patients’ informed consent. Specimens were immediately frozen and stored at -80℃ before western blot analyses.

**Quantitative RT-PCR**

Total RNA was extracted using TRIzol reagent (Invitrogen) and 1 μg total RNA was performed reverse transcription using PrimeScript RT reagent kit with gDNA eraser (TaKaRa), according to the manufacturer’s instructions. Quantitative RT-PCR was performed with SYBR Green dye using (Applied Biosystems). The relative amount of cDNA was calculated by the comparative Ct method using GAPDH as a control. PCR reactions were performed in triplicate.

**Western blot**

Protein extracts were prepared using NP-40 lysis buffer containing phosphatase and protease inhibitors, the protein concentration of the cell lysates were determined by BCA protein assay kit. The cell lysates were then subjected to SDS-PAGE followed by immunoblot using indicated antibodies.

**Extraction of cytoplasmic and nuclear proteins**

For the extraction of cytoplasmic and nuclear proteins, we used the Nuclear and Cytoplasmic Extraction Kit (CWBIO Cat. #CW0199S) according to the manufacturer’s protocol.

**Chromatin immunoprecipitation assay**

The ChIP-IT Express Magnetic Chromatin Immunoprecipitation Kit ＆ Sonication Shearing Kit (catalog nos. 53008 ＆ 53032) purchased from Active Motif were used in this experiment.

Cells were fixed with 1% formaldehyde for 10 min at room temperature for fixation. Then stop the fixation by adding Glycine Stop-Fix Solution. Cells were collected and lysed in lysis buffer and Shearing Buffer (supplemented with PIC and PMSF) at 4 ℃ and sonicated to disrupt DNA (200–1000 bp), Ten percent of the sample was kept as input. The sonicated DNA fragments were then diluted in a buffer comprising ChIP Buffer 1, Protease Inhibitor Cocktail (PIC), dH_2_O, rabbit His-tag antibody (CST: 12698), control rabbit IgG incubated with Protein G Magnetic Beads for 6 hours at 4 ℃. The beads were washed with ChIP Buffer 1 and ChIP Buffer 2. The beads were eluted with Elution Buffer AM2. Cross-linking was reversed by adding Reverse Cross-linking Buffer and incubating at 95 ℃ for 15 minutes. Treated with Proteinase K at 37 ℃ for 1 hour. Added Proteinase K Stop Solution. Then the DNA was analyzed by PCR using the following primers:

ANAPC13:

5′- TCCCTAAGCACAGAGCATGG-3′ (sense)

5′- AGTGTCGGACTTCCCCCTAT-3′ (antisense)

RAD21:

5′-TGGCTCTATAGGGATGAAAGT-3′ (sense)

5′-TAGATTGCTCTGGGTAGTATGG-3′ (antisense)

CDC7:

5′-TCACTGGCACAAACAGGTCC-3′ (sense)

5′-TCTTTAGGGTCAGACCAGTGC-3′ (antisense)

CDC27:

5′-TAGTAGAGACAGGGTTTCACTG-3′ (sense)

5′-TTATTCTGGCTGTAGATGAGG-3′ (antisense)

**Immunofluorescence staining**

The cells were cultured in 12 well plates for 18-24 h, fixed with 100% methanol for 20 min at room temperature and washed three times with 1×PBS. Then the cells were treated with 0.1% TritonX-100 (Sigma T8787-50) for 5 min at room temperature and blocked with 2.5% BSA for 1 h. The cells were then incubated with the indicator antibody at room temperature for 1 hour. After washing with 1 × PBS, the cells were incubated with rhodamine binding secondary antibody (Protentech) for 1 h in the dark at room temperature. After that, the cells were washed three times with 1 × PBS and were mounted with DAPI Fluoromount-G mounting medium (SouthernBiotech, 0100–20). DAPI Fluoromount-G was used to stain the nuclei. Immunofluorescence images were captured with Olympus IX83 inverted microscope and processed with Olympus CellSens™ Microscope Imaging Software.

**Immunohistochemistry**

Lung adenocarcinoma tissue microarray analysis was purchased from the Shanghai Outdo Biotech Co.,Ltd. The NSCLC patient samples (94 cases) were collected during 2004/9 to 2009/3. A detailed description of the human specimens is included in Supplementary Table 1. The expression of GRHL1 in the tissue was examined by immunohistochemical staining with an anti-GRHL1 antibody. This work was completed by Servicebio Co.,Ltd. The staining density was analyzed by GenePix software.

**Supplementary Figure legends**


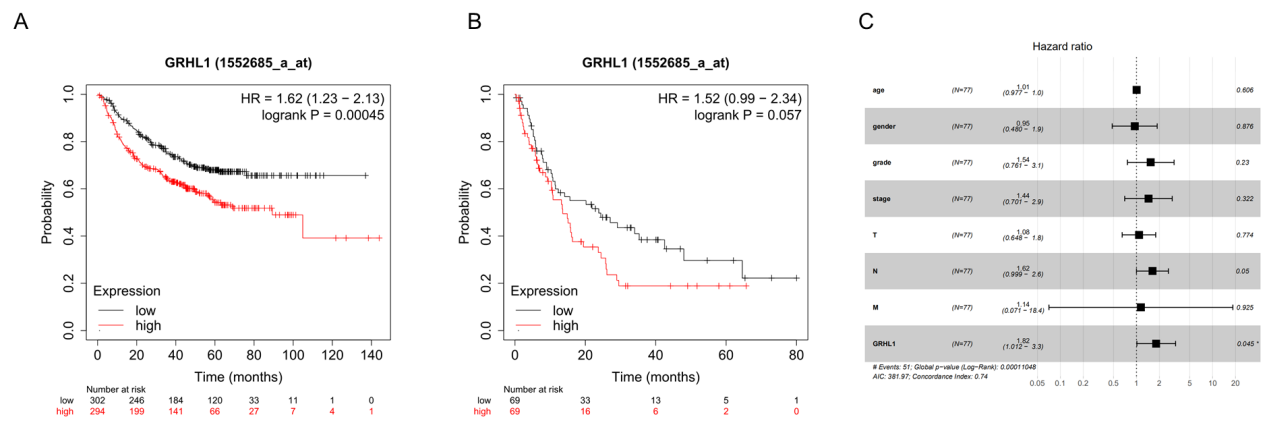


**Supplementary Figure 1**. GRHL1 is closely related to the poor survival of NSCLC patients. **A** The first progression survival curves of NSCLC patients were analyzed using Kaplan-Meier Plotter (https://kmplot.com/analysis/). **B** The post progression survival curves of NSCLC patients were analyzed using Kaplan-Meier Plotter (https://kmplot.com/analysis/). **C** The data of tissue microarray (after deleting cases with missing data) were used to judge whether the expression level of GRHL1 can be independent of the factors such as age, gender, classification, and T, N, M stages to predict the prognosis of patients by multivariate COX regression analysis.


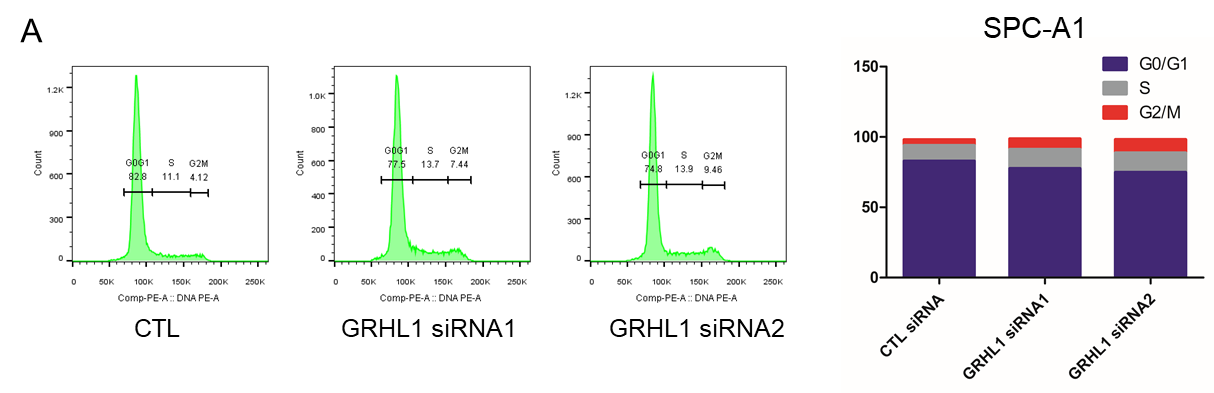


**Supplementary Figure 2**. GRHL1 knockdown arrests the cell cycle in the G2/M phase. **A** SPC-A1 cells were transfected with either control (CTL) siRNA or GRHL1 siRNAs. After 24 h, cell cycle analysis was done by flow cytometry. The inserts show the quantification of the cell cycle analysis.


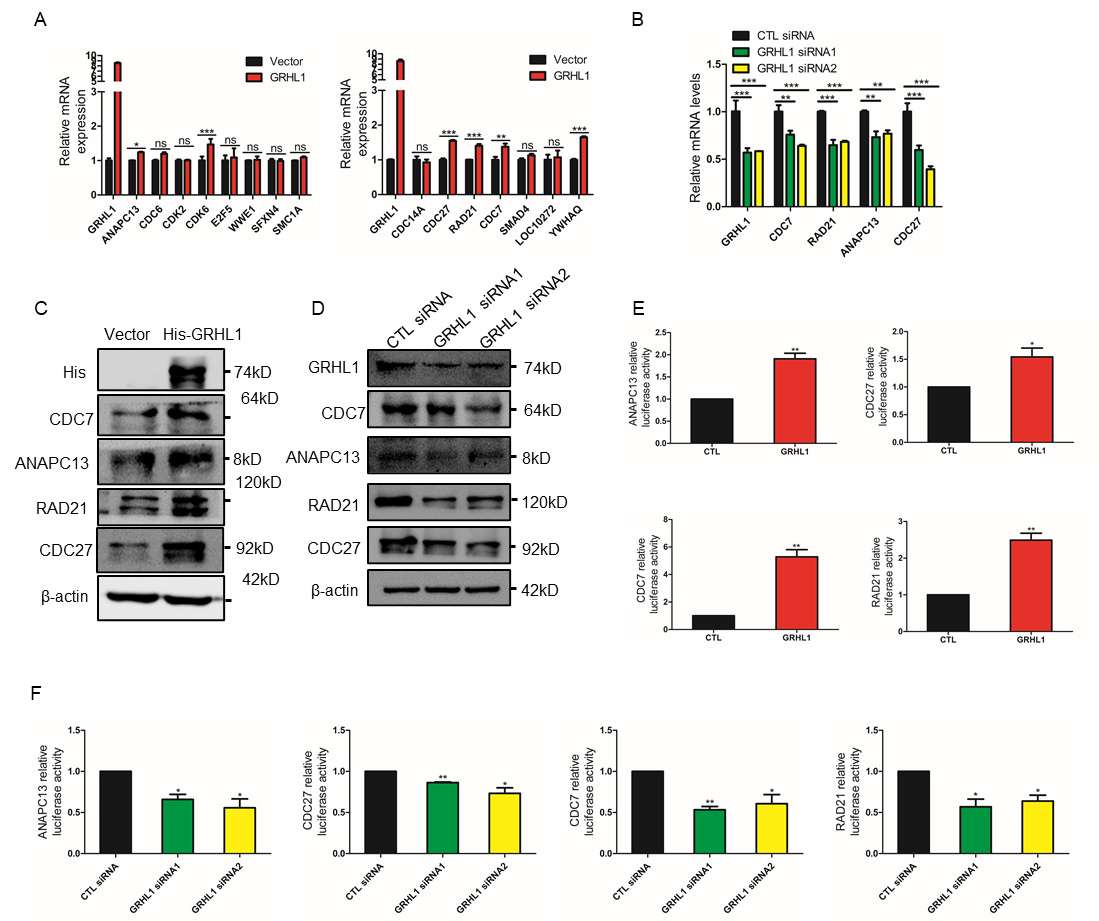


**Supplementary Figure 3**. GRHL1 regulates the transcription of genes in the G2/M phase. **A** A549 cells were transiently transfected with control or GRHL1 plasmids. Forty-eight hours later, total RNAs were extracted. The mRNA levels were determined by q-PCR. *P < 0.05, **P < 0.01, ***P < 0.001, ns: P > 0.05. **B** A549 cells were transiently transfected with control or GRHL1 siRNAs. Forty-eight hours later, total RNAs were extracted. The mRNA levels were determined by q-PCR. **P < 0.01, ***P < 0.001. **C** A549 cells were transiently transfected with control or His-GRHL1 plasmids. Forty-eight hours later, the cells were lysed. Protein expression was assessed by western blotting using the indicated antibodies. **D** A549 cells were transiently transfected with control or GRHL1 siRNAs. Forty-eight hours later, the cells were lysed. Protein expression was assessed by western blotting using the indicated antibodies. **E** pGL3-enhancer vector containing CDC27, RAD21, CDC7, and ANAPC13 promoter fragment was transfected into A549 cells respectively, co-transfected with Renilla control plasmid and pcDNA3.1 vector or pcDNA3.1-GRHL1 plasmid. The relative levels of luciferase activity were normalized to the levels of vector control and to the levels of luciferase activity of the Renilla control plasmid. Data represent the average of three independent experiments (mean ± SD). *P < 0.05, **P < 0.01. **F** pGL3-enhancer vector containing CDC27, RAD21, CDC7, and ANAPC13 promoter fragment was transfected into A549 cells respectively, co-transfected with Renilla control plasmid and control siRNA or GRHL1 siRNAs. The relative levels of luciferase activity were normalized to the levels of vector control and the levels of luciferase activity of the Renilla control plasmid. Data represent the average of three independent experiments (mean ± SD). *P < 0.05, **P < 0.01.


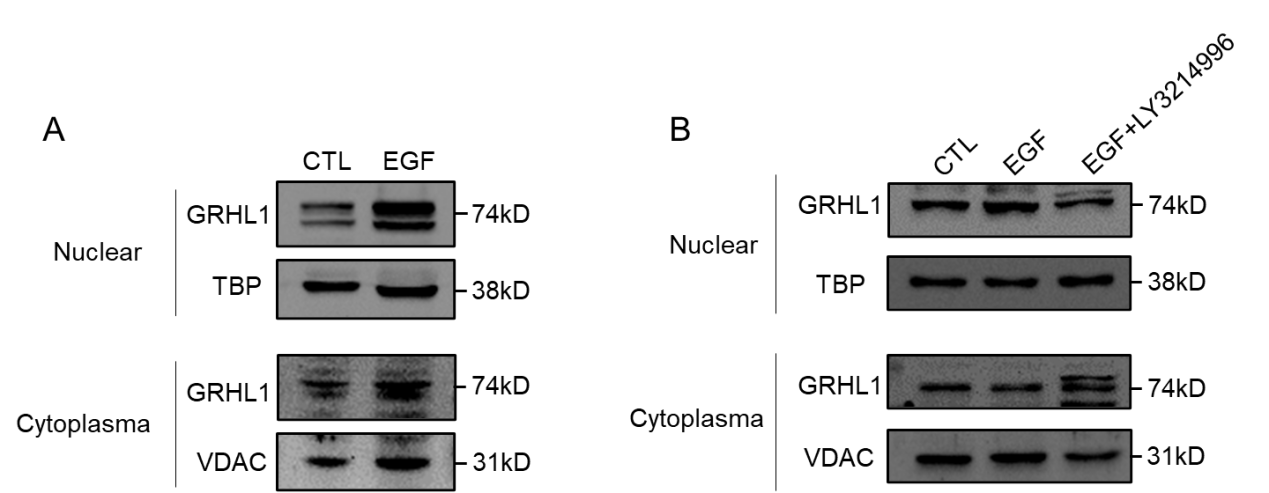


**Supplementary Figure 4**. EGFR-ERK pathway activates GRHL1 and promotes its nuclear translocation. **A** After 24 hours of stimulation with EGF (100 ng/ml), the nuclear and cytoplasmic fractions were separated and the location of GRHL1 was detected by western blot in H1299 cells. TBP: TATA binding protein, VDAC: mitochondrial outer membrane protein porin. **B** After serum-free treatment for 12 hours of H1299 cells, LY3214996 (ERK inhibitor, 300 nM) was added. 12 hours later, EGF (100 ng/ml) was added to both two experimental groups. After 24 hours, the nuclear and cytoplasmic fractions were separated and the location of GRHL1 was detected by western blot.


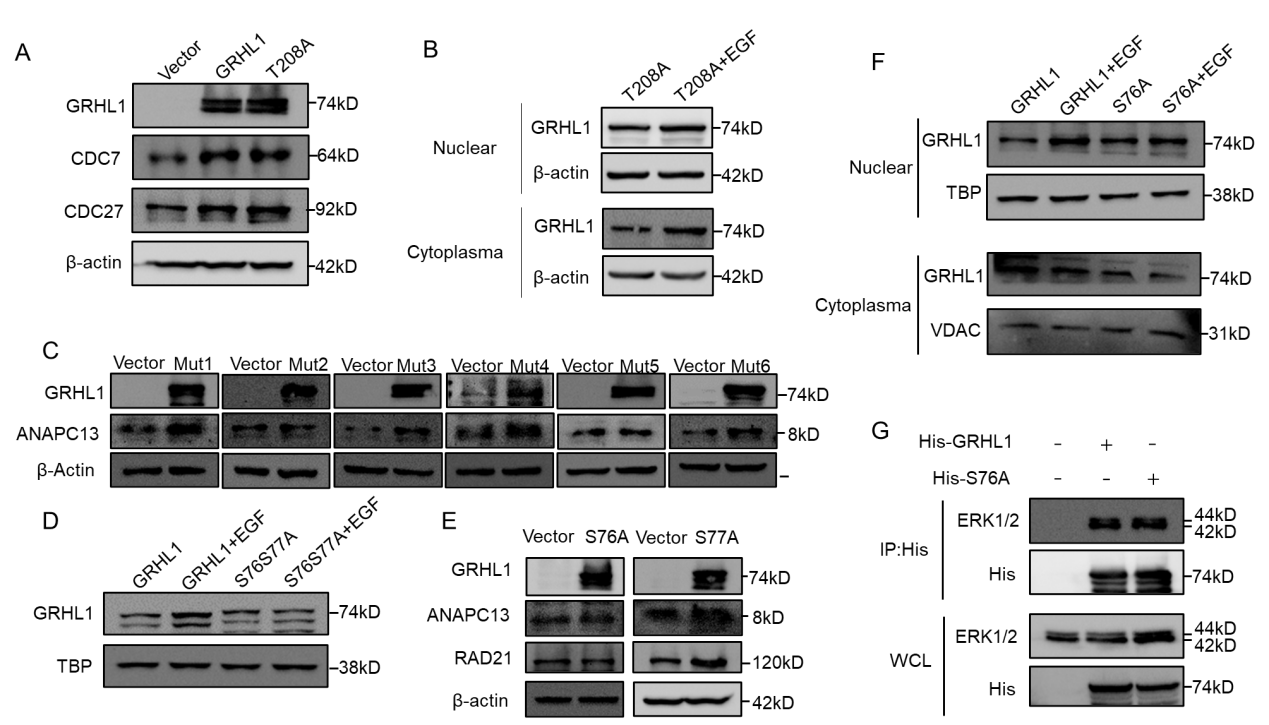


**Supplementary Figure 5**. ERK is responsible for the phosphorylation of GRHL1 at Ser76. **A** H1299 cells were transiently transfected with a control plasmid, His-GRHL1 plasmid, and His-GRHL1(T208A) plasmid respectively. Forty-eight hours later, the cells were lysed. Protein expression was assessed by western blotting using the indicated antibodies. **B** H1299 cells were transfected with His-GRHL1 plasmid and His-GRHL1(T208A) plasmid, after serum-free treatment for 12 hours and 24 hours of stimulation with EGF (100 ng/ml), the nuclear and cytoplasmic fractions were separated and the location of GRHL1 was detected by western blot. **C** H1299 cells were transiently transfected with control plasmid and six mutants (mut1: S25S28S35A, mut2: S76S77A, mut3: S95A, mut4: S442A, mut5: S493S501A, mut6: S539A) plasmids respectively. Forty-eight hours later, the cells were lysed. Protein expression was assessed by western blotting using the indicated antibodies. **D** H1299 cells were transfected with His-GRHL1 plasmid and His-GRHL1(S76S77A) plasmid, after serum-free treatment for 12 hours and 24 hours of stimulation with EGF (100 ng/ml), the nuclear and cytoplasmic fractions were separated and the GRHL1 in nuclear was detected by western blot. **E** H1299 cells were transiently transfected with a control plasmid, His-GRHL1(S76A), and His-GRHL1(S77A) plasmids respectively. Forty-eight hours later, the cells were lysed. Protein expression was assessed by western blotting using the indicated antibodies. **F** H1299 cells were transfected with His-GRHL1 plasmid and His-GRHL1(S76A) plasmid, after serum-free treatment for 12 hours and 24 hours of stimulation with EGF (100 ng/ml), the nuclear and cytoplasmic fractions were separated and the location of GRHL1 was detected by western blot. **G** 293T cells transfected with a control plasmid, His-GRHL1 plasmid, and His-GRHL1(S76A) plasmid respectively, after 48 hours, cells were lysed for immunoprecipitation using an anti-His antibody and blotted with indicated antibodies.

**Supplementary Table 1.** The detailed description of the human specimens in Lung adenocarcinoma tissue microarray.

**Supplementary Table 2.** The mass-spectrometry analysis for identification of the phosphorylation site in GRHL1.
